# Dietary fish, n-3 polyunsaturated fatty acid consumption, and depression risk in Japan: a population-based prospective cohort study

**DOI:** 10.1038/tp.2017.206

**Published:** 2017-09-26

**Authors:** Y J Matsuoka, N Sawada, M Mimura, R Shikimoto, S Nozaki, K Hamazaki, Y Uchitomi, S Tsugane

**Affiliations:** 1 Division of Health Care Research, QOL Research Group, Center for Public Health Science, National Cancer Center Japan, Chuo-ku, Tokyo, Japan; 2 Innovation Center for Supportive, Palliative and Psychosocial Care and Department of Psycho-Oncology, National Cancer Center Hospital, Chuo-ku, Tokyo, Japan; 3 Epidemiology and Prevention Group, Center for Public Health Science, National Cancer Center Japan, Chuo-ku, Tokyo, Japan; 4 Department of Neuropsychiatry, Keio University School of Medicine, Shinjuku-ku, Tokyo, Japan; 5 Department of Public Health, Faculty of Medicine, University of Toyama, Toyama, Toyama, Japan; 6 QOL Research Group, Center for Public Health Science, National Cancer Center Japan, Chuo-ku, Tokyo, Japan

## Abstract

Systematic review of observational studies has revealed that fish consumption and levels of n-3 polyunsaturated fatty acids (PUFAs) such as eicosapentaenoic acid (EPA) and docosahexaenoic acid are associated with a reduced risk of depression. A reverse J-shaped effect of n-3 PUFAs was suggested. However, there is limited evidence from populations with high fish consumption and no studies have used a standard psychiatrist-based diagnosis of major depressive disorder (MDD). Therefore, this population-based, prospective study investigated the association of dietary fish, n-3 PUFA, and n-6 PUFA consumption with risk of psychiatrist-diagnosed MDD in Japan. A total of 12 219 subjects were enrolled from the Saku area in 1990. Of these, we extracted 1181 participants aged 63–82 years who completed food frequency questionnaires in both 1995 and 2000 and also underwent a mental health examination in 2014–2015. Odds ratios (ORs) and 95% confidence intervals (CIs) for MDD according to fish intake and PUFA quartiles were calculated. Current MDD was diagnosed in 95 patients. We found a reduced risk of MDD in the third quartile for fish intake (111.1 g per day, OR=0.44, 95% CI=0.23–0.84), second quartile for EPA (307.7 mg per day, OR=0.54, 95% CI=0.30–0.99) and third quartile for docosapentaenoic acid (DPA) (123.1 mg per day, OR=0.42, 95% CI=0.22–0.85). ORs adjusted for cancer, stroke, myocardial infarction and diabetes remained significant for fish and DPA intake. Our results suggest that moderate fish intake could be recommended for the prevention of MDD in aged Japanese individuals.

## Introduction

An early ecological study showing a significant inverse correlation between per capita fish consumption, that is, primary dietary sources of eicosapentaenoic acid (EPA) and docosahexaenoic acid (DHA), and 1-year prevalence of depression^1^ created new interest in the link between depression and n-3 polyunsaturated fatty acids (PUFAs). One meta-analysis found low peripheral levels of n-3 PUFAs, EPA and DHA in patients with depression compared with controls.^2^ In addition, the magnitude of the effect size of differences in the levels of n-3 PUFAs was larger in analyses restricted to studies that used DSM criteria for major depressive disorder (MDD). Several recent meta-analyses of clinical trials indicated that n-3 PUFA supplementation has a beneficial effect in patients with depression^3, 4, 5, 6^ and showed that EPA-predominant formulations are more efficacious than placebo for the treatment of DSM-diagnosed MDD.^6^ The therapeutic mechanism of n-3 PUFAs remains unclear, but it is assumed that n-3 PUFAs have diverse neurobiological activities related to immunomodulation, anti-inflammation, neurotransmission and neuroprotection, contributing to their anti-depressive effects.^7^

Based on these previous findings, it is expected that n-3 PUFA intake will be beneficial for preventing depression. Although previous studies in healthy populations with mild depressive symptoms failed to show that ~2.0 g per day n-3 PUFA supplementation could prevent depression,^4^ a recent meta-analysis of 31 observational studies involving 255 076 individuals and more than 20 000 patients with depression supported the hypothetical association between intake of fish and n-3 PUFAs and decreased risk of depression.^8^ In this recent study, a decreased risk was found for 50 g per day of fish, 1.8 g per day of n-3 PUFAs and 0.6 g per day of EPA+DHA, even though a nonsignificant decreased risk was observed for higher intake of fish and EPA+DHA.^8^ Dietary intake of n-3 PUFAs is three to four times higher in Japan than in Western countries.^9^ As only 3% of the meta-analysis population in Grosso *et al.*^8^ was Japanese, more evidence from the Japanese population is needed. Furthermore, only 1 of the 31 studies assessed MDD by using a structured clinical interview for DSM and no study assessed MDD diagnosed by a psychiatrist. An important question is what dose of fish or n-3 PUFA intake reduces the risk of MDD development in the Japanese population, who frequently eat fish.

To address the hypothesis that high fish or n-3 PUFA intake prevents MDD even in individuals from a fish-eating culture, we examined the association between fish or n-3 PUFA intake and psychiatrist-diagnosed MDD in a population cohort of Japanese men and women. This information would be useful for the prevention of MDD in Japan as well as in other countries with a fish-eating population.

## Materials and methods

### Study population

**Figure 1:**
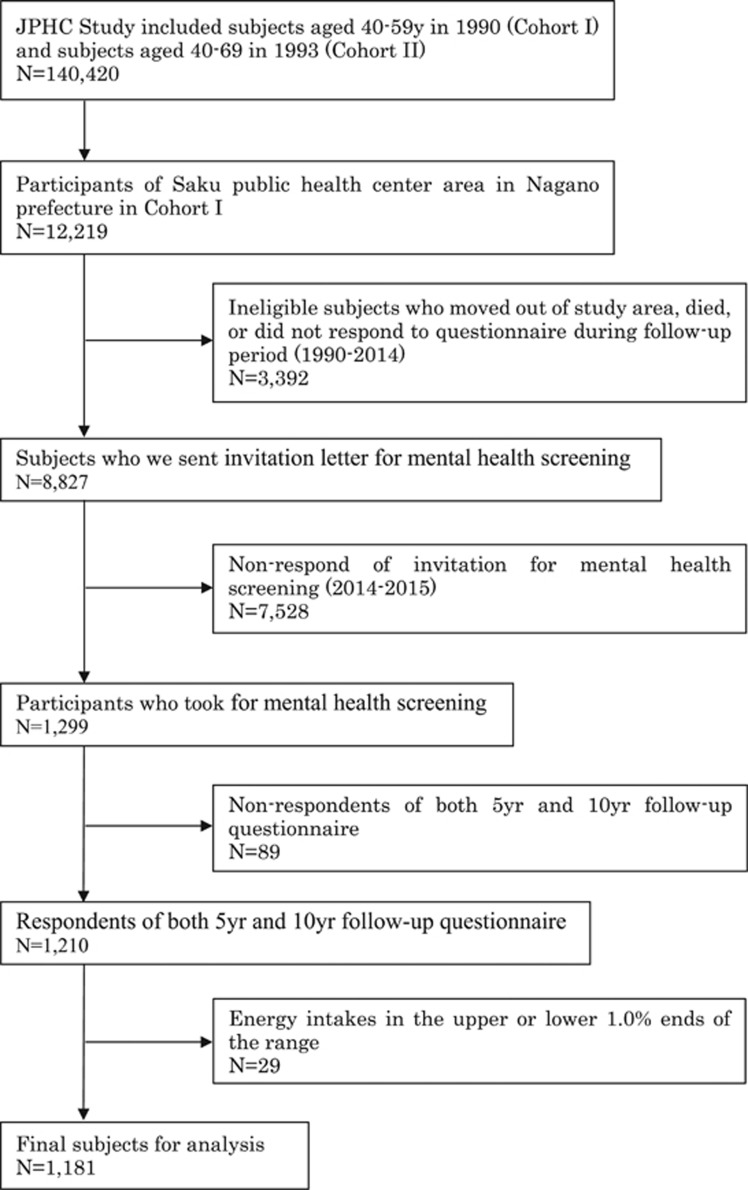
Flow diagram of the study population.

This study is a secondary data analysis of the Japan Public Health Center-based Prospective Study (JPHC Study) that commenced in 1990 for cohort I and in 1993 for cohort II.^10^ The study was approved by the Institutional Review Board of the National Cancer Center Japan and Keio University School of Medicine. In the JPHC Study, which is described in detail elsewhere,^10^ we distributed a questionnaire at baseline, at the 5-year follow-up, and again at the 10-year follow-up (response rate, 74–81%). As shown in Figure 1, the present study population comprised residents in the Saku Public Health Center catchment area (Nagano prefecture) from the 1990 cohort, which involved 12 219 participants (6172 men, 6047 women) aged 40–59 years at the beginning of the study. After excluding participants who subsequently moved out of the study area, died, or did not respond to the later questionnaires, we posted an invitation letter for mental health screening to 8827 participants in 2014–2015. Of the 1299 who responded to the call for screening, 1210 had completed the dietary assessment in both 1995 (5-year follow-up survey) and 2000 (10-year follow-up survey). We excluded participants (*n*=29) who reported extreme energy intake (calculated using both the 1995 and 2000 questionnaires) at the upper or lower 1.0% end of the range (1284 and 4279 kcal for men and 1033 and 4419 kcal for women, respectively), because extreme data tend to be inaccurate. This left 1181 participants (491 men, 690 women) for the final analysis. Participants provided written informed consent to take part in a mental health screening survey in 2014–2015.

### Dietary assessment

A food frequency questionnaire was used at both the 5-year and 10-year follow-up surveys to assess the intake of 147 food items, including 19 fish and shellfish items. Participants were asked how often during the previous year had they consumed each food item. As described in a previous study,^11^ standard portion sizes for each item were classed as small (50% less than standard), medium (same as standard) and large (50% more than standard). There were nine frequency categories for each item (never, 1–3 times per month, 1–2 times per week, 3–4 times per week, once per day, 2–3 times per day, 4–6 times per day and ⩾7 times per day). The 19 items that concerned fish or shellfish were salted fish, dried fish, canned tuna, salmon or trout, bonito or tuna, cod or flat fish, sea bream, horse mackerel or sardine, mackerel pike or mackerel, dried small fish, salted roe, eel, octopus, prawn, short-necked clam, crab shell, vivipara, *chikuwa* (a fish paste product) and *kamaboko* (a fish paste product). Daily fish consumption (g per day) was calculated by multiplying the frequency by the standard portion size for each food item. The fatty acid composition table of Japanese foods^12^ was used to calculate the daily intake of n-3 PUFAs and n-3 PUFA subtypes (i.e., alpha-linolenic acid (ALA), EPA, docosapentaenoic acid (DPA) and DHA) and the daily intake of the n-6 PUFAs linoleic acid (LA) and arachidonic acid (AA). Average intake was calculated by using the same questionnaire in both the 5-year and 10-year follow-up surveys. Food and nutrient intake was log-transformed and adjusted for total energy intake using the residual model.^13^ The validity of the food frequency questionnaire in the assessment of fish, ALA, EPA, DPA and DHA consumption in subsamples was confirmed using 14- or 28-day dietary records. Spearman’s correlation coefficients between the energy-adjusted intake of fish, n-3 PUFAs, ALA, EPA, DPA, DHA, n-6 PUFAs, LA and AA calculated from the questionnaire and from dietary records were 0.20, 0.21, 0.27, 0.38, 0.32, 0.34, 0.30, 0.29 and 0.32 for men and 0.25, 0.34, 0.25, 0.45, 0.39, 0.37, 0.21, 0.22 and 0.25 for women, respectively,^14, 15^ indicating moderate validity for fish and n-3 PUFAs.

### Follow-up and identification of physical illness and depression

Subjects in the Saku area were followed up until the present screening in 2014–2015. Any changes in residence, including death, were determined annually using the residential registry in the catchment area. Incidence data on cancer were identified from local major hospitals in the study area.^10^ Although we registered stroke and myocardial infarction incidence from local major hospitals, their registration finished in 2009. Therefore, history of stroke, myocardial infarction, diabetes mellitus and depression was identified by questionnaire at the mental health screening.

### Current psychiatric assessment

We administered the Center for Epidemiological Scale-Depression (CES-D)^16, 17^ and the Patient Health Questionnaire-9 (PHQ-9)^18, 19^ screening tests simultaneously at the mental health screening. Then, each participant was assessed by a trained psychiatrist irrespective of their CES-D and PHQ-9 scores. CES-D and PHQ-9 scores were presented to a psychiatrist at the clinical interview. Finally, trained psychiatrists assessed whether the participants currently met the DSM-IV criteria for MDD after considering if their depressive symptoms caused clinically significant distress or impairment. We did not assess the inter-rater reliability for the current major depressive episode.

### Statistical analysis

We used logistic regression analyses to calculate odds ratios (ORs) and 95% confidence intervals (CIs) for current MDD diagnosis per quartile consumption of fish, n-3 PUFAs, EPA, DHA, EPA+DHA, DPA, ALA, n-6 PUFAs, LA and AA and the n-3/n-6 PUFA ratio compared with the lowest consumption category as reference. OR estimates were adjusted for age and sex. Then, a multivariate model was adjusted for potential confounding variables: age, sex, smoking status (never, former, current), alcohol consumption frequency (almost never, 1–3 times per month, ⩾1 times per week), physical activity (continuous, metabolic equivalents of task (METs)), history of depression (yes or no), history of cancer (yes or no), history of stroke (yes or no), history of myocardial infarction (yes or no) and history of diabetes mellitus (yes or no). We tested trends across quartiles for intake of n-3 PUFAs, EPA, DHA, EPA+DHA, DPA, ALA, n-6 PUFAs, LA and AA, and the n-3/n-6 PUFA ratio using ordinal numbers (0–3) assigned to quartile categories. All *P*-values were two-sided and statistical significance was set at *P*<0.05. All statistical analyses were performed with SAS software version 9.1 (SAS Institute, Cary, NC, USA).

## Results

A total of 99 participants were estimated to have current MDD on the basis of a CES-D score ⩾16 or PHQ-9 score ⩾10. Finally, a total of 95 participants were diagnosed with current MDD by a trained psychiatrist. Mean score of CES-D and PHQ-9 in MDD cases was 19.1 (s.d.=4.5) and 7.5 (s.d.=3.4), respectively. Table 1 shows participant characteristics according to total fish consumption at the final screening in 2014 and 2015 and at the follow-up survey in 2005. Mean participant age was 73 years and almost 60% were women. A significant difference was found in physical activity, with participants with a higher intake of fish tending to be less physically active. The PUFA intake pattern was similar to that seen for fish intake. Thus, participants with higher intake of fish tended to consume both n-3 PUFAs and n-6 PUFAs.

Table 2 presents the association of the quartiles for n-3 PUFAs, EPA, DHA, EPA+DHA, DPA, ALA, n-6 PUFAs, LA, and AA, and the n-3/n-6 PUFA ratio with MDD. Total fish consumption, EPA and DPA had a reverse J-shaped association with the risk of MDD. There was significantly reduced risk in the third quartile for fish intake (111.1 g per day, OR=0.44, 95% CI=0.23–0.84), second quartile for EPA intake (307.7 mg per day, OR=0.54, 95% CI=0.30–0.99) and third quartile for DPA intake (123.1 mg per day, OR=0.42, 95% CI=0.22–0.81). ORs adjusted for cancer, stroke, myocardial infarction and diabetes remained significant for fish and DPA intake. We found no significant association of the intake of total n-3 PUFAs, DHA, EPA+DHA, ALA, n-6 PUFAs, LA or AA, or the n-3/n-6 ratio with MDD.

## Discussion

In this population-based prospective cohort study, we tested the hypothesis that high intake of fish or n-3 PUFAs prevents MDD even in a Japanese population with a fish-eating culture. We did not find a simple linear association but instead a reverse J-shaped association of intake of fish, EPA or DPA with MDD. The results showed a decreased risk of MDD in those with a median intake of 111 g per day of fish, 307 mg per day of EPA, or 123 mg per day of DPA. To the best of our knowledge, this study is the first to examine the association of fish, n-3 PUFA, or n-6 PUFA consumption with a risk of later MDD diagnosis by psychiatrists via a longitudinal design with a long (25-year) follow-up in population-based individuals. Our findings are in line with those of a recent meta-analysis suggesting that dietary fish or n-3 PUFA intake is associated with a lower risk of depression.^8^

Six observational studies have investigated the association of fish and n-3 PUFA intake with depression in junior high school students,^20^ pregnant women,^21, 22, 23^ municipal employees^24^ and newly diagnosed lung cancer patients^25^ in Japan. A cross-sectional study by Murakami *et al.*^20^ showed that higher intakes of fish, EPA and DHA were associated with a lower prevalence of depressive symptoms in young adolescents. The cross-sectional study by Miyake *et al.*^22^ showed that higher intake of fish, EPA and DHA was associated with a lower prevalence of depressive symptoms during pregnancy. Another cross-sectional study by Suzuki *et al.*^25^ reported that ALA and total n-3 PUFA intake might be associated with lower prevalence of depressive symptoms. There was no information on the preventive value of fish or n-3 PUFAs regarding depression in the general older population. The present study adds important information not only to the field in general, but also specifically regarding the older Japanese population. In addition, the associations seen with fish and DPA intake remained significant after controlling for physical illness such as cancer, stroke, myocardial infarction and diabetes mellitus. Thus, fish intake might be one strategy for the prevention of MDD.

A reverse J-shaped effect of fish, EPA and DPA intake on decreased risk of MDD is in line with the findings of a recent meta-analysis.^8^ The exact reason for the non-linear relationship is not known, but the intake of other nutrients might counteract the effects of fish and n-3 PUFAs on depression. Indeed, n-6 PUFA intake gradually increased with an increase in fish intake. Total n-3 PUFA content in fish products varies considerably depending on fish species, feed management and food processing. For example, compared with pure fish fillets, breaded and pre-fried Alaska Pollock fillets contain extraordinarily high fat and n-6 PUFA levels.^26^ Because subjects with high fish consumption tended to eat more vegetables in the JPHC study,^27^ food processing such as stir-frying of vegetables could increase n-6 PUFA levels. However, the results remained significant after the addition of n-6 PUFA consumption as a covariate (data not shown). Furthermore, it might be possible that the participants at risk tried to consume more fish or n-3 PUFA to prevent depression. The optimal intake of fish for a reduced risk of depression in the present study (111 g per day) was double that of the meta-analysis. However, despite using a validated food frequency questionnaire, it is difficult to accurately estimate optimal intake. The National Health and Nutrition Examination Survey in 2013 reported that Japanese people over the age of 20 consumed median 66.0 g fish per day.^28^ It is not so difficult to eat 100 g fish in Japan. For example, ~100 g of grilled salmon is a popular breakfast option even in casual dining restaurants. Because there was no clear association between n-6 PUFAs and MDD in this study, a fish intake of 100 g per day might be enough to protect older Japanese individuals against MDD.

We already know the postulated mechanisms of the anti-depressive effects of EPA, such as anti-inflammation,^7^ but very little is known about DPA. DPA [22:5n-3] is an elongated metabolite of EPA and an intermediate in the biosynthesis of DHA.^29^ Serhan showed that n-3 PUFA-derived lipid mediators are involved in inflammation protection as well as in pain alleviation, host defense and organ protection from ischemia-reperfusion damage.^30^ They are called specialized pro-resolving mediators (SPMs). Recently, new SPMs, PD1_n-3 DPA_ and MaR1_n-3 DPA_ biosynthesized from the n-3 PUFA DPA, have been reported and their bioactions discussed.^31^ These new SPMs might possibly have an anti-depressive action by protecting against inflammation.

Several limitations should be mentioned. First, we estimated fish and n-3 and n-6 PUFA intake from a self-report questionnaire, which would not fully reflect tissue composition. Second, there may have been a selection bias, with 14% of Saku residents participating in the mental health screening in 2014 and 2015. Third, the findings may not necessarily apply to all nationalities and ethnicities. A previous cross-sectional study suggested that the associations of fish consumption with depression in large samples of older adults in seven low- and middle-income countries varied markedly across countries and could be explained by socio-demographic and lifestyle variables.^32^ Fourth, we did not administer a structured clinical interview for psychiatric diagnosis, but assessed the participants clinically, which may somewhat weaken the reliability. Fifth, we cannot rule out the possibility that our findings might be based on chance due to many exposure variables and the relatively small number of cases. Finally, because the subjects in this study were older (mean age, 73 years), some participants might have had mild cognitive impairment, which was not excluded from the present study. Despite these limitations, the repeated use of the same questionnaire was a major advantage in our study. Even if our results were affected by misclassification due to a changing dietary pattern as a result of the food frequency questionnaire, such misclassification would probably be non-differential and would underestimate the true relative risk.

In conclusion, our population-based prospective cohort study indicated that an estimated intake of 111 g per day of fish, 307 mg per day of EPA or 123 mg per day of DPA was associated with a reduced risk of MDD in a Japanese population with a fish consumption culture. Emerging and compelling evidence suggests that diet and nutrition are extremely important factors in the high prevalence of depressive disorders,^33^ and our findings provide a basis to further examine the effectiveness of fish and n-3 PUFA intake for the prevention of MDD in both aged individuals.

## Disclaimer

The views expressed in this article are those of the authors and do not necessarily represent the views of the National Cancer Center Japan.

## Figures and Tables

**Table 1:** Subject characteristics according to fish consumption (*n*=1181)

	*Total fish consumption*				
	*Lowest*	*Second*	*Third*	*Highest*	P_*difference*_
Median (g per day)	57.2	83.8	111.1	152.6	
					
*Information at screening*					
Age, years±s.d.	72.7±5.5	73.3±5.6	73.6±5.6	73.5±5.8	0.23
Male (%)	43.6	39.3	39.2	44.1	0.46
History of depression (yes), %	2.0	2.7	1.1	2.7	0.46
History of diabetes (yes), %	9.2	11.9	10.1	8.1	0.47
History of cancer (yes), %	12.5	12.2	14.7	10.8	0.56
History of stroke (yes), %	3.3	4.8	4.6	3.4	0.72
History of myocardial infarction (yes), %	2.0	1.0	1.4	3.4	0.18
					
*Information from 2005 survey*					
Current smoker, %	20.8	14.6	15.4	20.9	0.07
Regular drinker (yes), %	28.4	29.2	27.3	25.6	0.88
Physical activity (METs), mean±s.d.	39.3±9.5	37.8±9.7	36.9±10.1	36.8±9.5	<0.01
n-3 PUFA, mean±s.d. (g per day)	2.3±0.5	2.8±0.5	3.2±0.5	3.9±0.7	<0.0001
Eicosapentaenoic acid, mean±s.d. (mg per day)	199.1±61.8	316.3±55.7	418.3±78.4	603.6±167.9	<0.0001
Docosahexaenoic acid, mean±s.d. (mg per day)	363.1±112.6	551.5±91.7	718.6±129.3	1013.0±262.0	<0.0001
Docosapentaenoic acid, mean±s.d. (mg per day)	67.5±18.8	97.7±16.3	125.7±23.0	173.8±43.3	<0.0001
Alpha-linoleic acid, mean±s.d. (mg per day)	1577.6±448.3	1693.3±400.4	1733.5±358.8	1812.4±342.7	<0.0001
n-6 PUFA, mean±s.d. (g per day)	11.5±2.6	12.2±2.6	12.4±2.1	12.8±2.1	<0.0001
Linoleic acid, mean±s.d. (g per day)	11.2±2.6	11.9±2.6	12.1±2.1	12.4±2.1	<0.0001

Abbreviations: MET, metabolic equivalents of task; PUFA, polyunsaturated fatty acid.

**Table 2:** Odds ratios and 95% confidence intervals for depression according to quartile of intake of fish and n-3 PUFA in the JPHC Study (*n*=1181)

	*Quartile*				
	*Lowest*	*Second*	*Third*	*Highest*	P_*trend*_
*Fish (median, g per day)*	57.2	83.8	111.1	152.6	
No. of cases/controls	32/271	23/272	15/271	25/272	
Age, sex-adjusted OR (95% CI)	1.00	0.68 (0.39–1.20)	0.44 (0.23–0.84)	0.74 (0.43–1.29)	0.15
Multivariate OR^a^ (95% CI)	1.00	0.66 (0.37–1.17)	0.44 (0.23–0.84)	0.73 (0.41–1.28)	0.15

*n-3 PUFA (median, g per day)*	2.2	2.8	3.3	3.9	
No. of cases/controls	27/271	25/272	20/271	23/272	
Age, sex-adjusted OR (95% CI)	1.00	0.85 (0.48–1.50)	0.66 (0.36–1.21)	0.76 (0.42–1.37)	0.26
Multivariate OR^a^ (95% CI)	1.00	0.86 (0.48–1.55)	0.69 (0.37–1.28)	0.77 (0.42–1.40)	0.30

*EPA (median, mg per day)*	199.9	307.7	404.5	577.9	
No. of cases/controls	31/271	18/272	20/271	26/272	
Age, sex-adjusted OR (95% CI)	1.00	0.54 (0.30–0.999)	0.61 (0.34–1.10)	0.79 (0.45–1.37)	0.45
Multivariate OR^a^ (95% CI)	1.00	0.55 (0.30–1.03)	0.65 (0.36–1.17)	0.78 (0.44–1.37)	0.48
					
*DHA (median, mg per day)*	363.3	534.0	704.2	969.9	
No. of cases/controls	29/271	21/272	19/271	26/272	
Age, sex-adjusted OR (95% CI)	1.00	0.68 (0.38–1.24)	0.62 (0.34–1.13)	0.85 (0.49–1.49)	0.52
Multivariate OR^a^ (95% CI)	1.00	0.66 (0.36–1.20)	0.64 (0.34–1.18)	0.84 (0.47–1.48)	0.54

*EPA+DHA (median, mg per day)*	561.1	843.1	1105.0	1555.2	
No. of cases/controls	29/271	21/272	19/271	26/272	
Age, sex-adjusted OR (95% CI)	1.00	0.68 (0.38–1.23)	0.62 (0.34–1.14)	0.84 (0.48–1.48)	0.51
Multivariate OR^a^ (95% CI)	1.00	0.68 (0.37–1.23)	0.66 (0.36–1.22)	0.84 (0.48–1.49)	0.55

*DPA (median, mg per day)*	67.1	95.90	123.1	169.4	
No. of cases/controls	31/271	22/272	14/271	28/272	
Age, sex-adjusted OR (95% CI)	1.00	0.69 (0.39–1.22)	0.42 (0.22–0.81)	0.87 (0.50–1.49)	0.37
Multivariate OR^a^ (95% CI)	1.00	0.68 (0.38–1.23)	0.44 (0.23–0.85)	0.85 (0.49–1.47)	0.36

*Alpha-linolenic acid (median, mg per day)*	1237.5	1573.4	1828.7	2159.8	
No. of cases/controls	22/271	20/272	28/271	25/272	
Age, sex-adjusted OR (95% CI)	1.00	0.87 (0.46–1.63)	1.17 (0.65–2.11)	1.02 (0.55–1.86)	0.72
Multivariate OR^a^ (95% CI)	1.00	0.90 (0.48–1.71)	1.17 (0.65–2.13)	1.06 (0.57–1.97)	0.65

*n-6 (median, g per day)*	9.5	11.6	12.8	14.7	
No. of cases/controls	21/271	23/272	25/271	26/272	
Age, sex-adjusted OR (95% CI)	1.00	1.04 (0.56–1.93)	1.11 (0.60–2.04)	1.10 (0.60–2.03)	0.72
Multivariate OR^a^ (95% CI)	1.00	1.03 (0.55–1.93)	1.07 (0.58–2.00)	1.12 (0.60–2.08)	0.70

*Linolenic acid (median, g per day)*	9.3	11.2	12.5	14.4	
No. of cases/controls	21/271	25/272	23/271	26/272	
Age, sex-adjusted OR (95% CI)	1.00	1.13 (0.61–2.07)	1.02 (0.55–1.89)	1.09 (0.59–2.01)	0.88
Multivariate OR^a^ (95% CI)	1.00	1.12 (0.60–2.08)	1.00 (0.53–1.89)	1.11 (0.59–2.06)	0.85

*Arachidonic acid (median, mg per day)*	122.6	158.4	188.5	228.5	
No. of cases/controls	29/271	24/272	21/271	21/272	
Age, sex-adjusted OR (95% CI)	1.00	0.80 (0.45–1.42)	0.68 (0.38–1.23)	0.72 (0.40–1.30)	0.22
Multivariate OR^a^ (95% CI)	1.00	0.82 (0.46–1.46)	0.71 (0.39–1.29)	0.72 (0.39–1.31)	0.23

*n-3/n-6 (median, ratio)*	0.20	0.23	0.26	0.31	
No. of cases/controls	27/271	22/272	22/271	24/272	
Age, sex-adjusted OR (95% CI)	1.00	0.80 (0.44–1.44)	0.78 (0.43–1.41)	0.84 (0.47–1.50)	0.56
Multivariate OR^a^ (95% CI)	1.00	0.81 (0.45–1.47)	0.82 (0.45–1.49)	0.86 (0.48–1.56)	0.65

Abbreviations: DHA, docosahexaenoic acid; DPA, docosapentaenoic acid; EPA, eicosapentaenoic acid; n-3 PUFA-rich fish, salmon or trout, sea bream, horse mackerel or sardine, mackerel pike or mackerel and eel; PUFA, polyunsaturated fatty acid.

a Adjusted for age, sex, smoking status, alcohol frequency, physical activity, past history of depression, cancer, stroke, miocardial infarction and diabetes mellitus.
